# Adaptive mentalization-based integrative therapy for severe personality disorder and mental illness: study protocol for a controlled longitudinal cohort study

**DOI:** 10.3389/fpsyt.2025.1685896

**Published:** 2026-01-06

**Authors:** Fleur E. van Kaam, Remco F. P. de Winter, Coriene ten Kate, Rosanne S. H. Kop, Merel M. Lelieveld, Rozemarijn van Duursen, Maarten van Westen, Anne-Laura van Harmelen, Thérèse van Amelsvoort, Jonas G. Weijers

**Affiliations:** 1 GGZ Rivierduinen, Institute for Mental Health Care, Leiden, Netherlands; 2 MHeNs School for Mental Health and Neuroscience, Maastricht University, Maastricht, Netherlands; 3 Vrije Universiteit Amsterdam, Amsterdam, Netherlands; 4 Altrecht, Institute for Mental Health Care, Utrecht, Netherlands; 5 Institute of Education and Child Studies, Leiden University, Leiden, Netherlands

**Keywords:** adaptive mentalization-based integrative therapy, mentalizing capacity, epistemic trust, reflective functioning, personality disorder, severe mental illness, experience sampling method

## Abstract

**Background:**

Personality Disorders that meet the criteria for a Severe Mental Illness (SMI-PD) tend to coincide with a greatly reduced quality of life and a lower life expectancy. Despite the numerous and complex needs of patients with SMI-PD, effective treatments for this group have not yet been established. Traditional treatments often fail to address the multitude of problems patients with SMI-PD struggle with and there is limited understanding about the treatment needs and effects. SMI-PD is associated with high economic costs to both the individual and society. Adaptive Mentalization-based Integrative Therapy (AMBIT) is a mentalization-based systematic approach designed to support teams in their care for people with multiple needs who distrust help. This study will investigate the effectiveness and cost-effectiveness of AMBIT in treating patients with SMI-PD.

**Methods:**

Patients aged 18 or older with SMI-PD receiving care at GGZ Rivierduinen and GGZ Altrecht (*n* = 160), mental health facilities in the Netherlands, will be recruited for this controlled naturalistic longitudinal cohort study. To assess treatment effectiveness and cost-effectiveness, self-report questionnaires will be administered and an electronic diary through the Experience Sampling Method will be used at three time points: at baseline (T0; start of treatment), and after 9 (T1) and 18 (T2) months of treatment. The questionnaires and diary will assess demographics, personality, social, and societal functioning, mentalizing capacity, epistemic mistrust, childhood trauma, attachment security, suicidality, and psychological resilience. A qualitative interview will be held at the end of treatment to evaluate treatment experiences. Additionally, therapists will complete questionnaires to evaluate the effectiveness of AMBIT. Data will be analysed using intention-to-treat multilevel regression analysis.

**Discussion:**

Little is known about the treatment needs of patients with SMI-PD and the effectiveness and cost-effectiveness of an AMBIT approach on this group of patients. The results of the present study will therefore contribute to the development of more tailored and effective psychological treatment for this complex group of patients.

**Trial Registration:**

Research Portal of CCMO Onderzoek met mensen, Trial Registration Number NL-010303, https://www.onderzoekmetmensen.nl/en/trial/57877, registered on June 27, 2025. OSF Registries, https://doi.org/10.17605/OSF.IO/QHYCW, preregistered on May 7, 2025.

## 1 Introduction

Much progress has been made in the treatment of personality disorders (PDs) (1–4), but less is known about what constitutes effective treatment for patients with PD who also meet the criteria for a severe mental illness (SMI). Delespaul and the Dutch Consensus group EPA (5) defined SMI-PD by the severity and complexity of symptoms, comorbidity - most commonly PTSD, depression, anxiety, and substance abuse -, and a history of therapeutic ruptures, in addition to meeting criteria of SMI, to distinguish them from PD and SMI (5–7).

Patients with SMI-PD experience problems pertaining to the ability to function in important areas of life, such as work, relationships, self-care and social activities (5–8). Moreover, these social problems tend to contribute to the persistence of PD symptoms. Consequently, because of the multitude of social and mental health problems, this group of patients reports a substantially reduced quality of life and lower life expectancy (6–8). Suicidality is an important cause of reduced life expectancy in patients with SMI and SMI-PD (9, 10). The combination of psychiatric distress, personal functioning difficulties, and interpersonal problems characterizing personality disorders, along with work, financial, and social challenges, complicates treatment for patients meeting SMI-PD criteria. Although SMI-PD affects a relatively small subset of patients, the associated high levels of healthcare utilization and occupational impairments can generate considerable economic strain on both individual and societal levels (11). Given the chronicity and multifaceted nature of SMI-PD, conventional mental healthcare systems frequently fail to adequately address the needs most commonly required by this group of patients and they frequently find themselves inadequately supported within the mental healthcare system (6–8, 12, 13).

Effective treatment for individuals with SMI-PD should address the social and interpersonal impairments that are often rooted in insecure attachment. Attachment is an innate survival mechanism, guiding infants to seek comfort from caregivers in times of distress and shaping their understanding of social environments (14, 15). Secure attachment relationships foster epistemic trust, which is the ability to recognize information from others as genuine and personally relevant. Epistemic trust enables social learning, allowing the individual to benefit from their social environment and to generalize this information to other contexts (14, 15). In contrast, insecure attachment, typically resulting from early adverse experiences, is associated with epistemic mistrust and impaired mentalizing, which impedes social learning. Patients with SMI-PD often struggle with epistemic trust due to adverse childhood experiences and attachment disruptions, leading to mistrust, poor communication, and social withdrawal; these interpersonal problems serve as maintaining factors for their mental health problems (6–8, 16–18). Evidence-based mono-therapies for PDs, such as dialectical behaviour therapy (DBT) (19), mentalization-based therapy (MBT) (20), schema therapy (ST) (21) and transference-focused therapy (TFP) (22), mainly focus on one aspect of personality pathology that patients with PD suffer from but do not comprehensively address the complex range of needs required by patients with SMI-PD. These treatments are often less effective due to the high levels of distress and complexity of problems in these patients. Furthermore, the involvement of multiple professionals across different domains (e.g., healthcare, legal, social services), which is often required in addition to treatment of personality pathology, frequently leads to fragmented or contradictory care, undermining trust and coordination and eventually the effectiveness of treatment.

SMI-PD patients are also often “hard to reach”, with only a small proportion seeking help. Those who do seek help may have had negative past experiences with caregivers or therapists who are often not fully equipped for the mentalization challenges that this group of patients poses, or face discouragement from other attachment figures, such as family members. As such, the patients’ history of trauma and insecure attachment may lead them to avoid or reject conventional treatment, responding with mistrust or hostility. This dynamic challenges therapists who may themselves struggle to maintain mentalization and emotional resilience, increasing the risk of therapeutic rupture. When such therapeutic rupture happens, the therapeutic relationship suffers, which reinforces patients’ mistrust in the mental health system (6, 23–28).

The mental health care costs for patients with SMI-PD are in general high as a result of the severity and pervasiveness of their mental health problems, and failure to recover from conventional treatment due to their attachment problems leading to repeated rejection of help (11, 13, 29). Besides a health-related burden, the individual patient with SMI-PD can accrue significant economic strain, debt, and liability, which can also affect their immediate family and society. These costs consist of direct healthcare costs (i.e. increased stress, or poor lifestyle choices) and of non-healthcare costs such as reduced productivity due to illness or limited societal participation (4, 30, 31). Cost-effectiveness is therefore, together with effectiveness, an important consideration in the choice of treatment. Health economic evaluations can contribute to decision making on how to optimally allocate (scarce) health care resources.

In the Netherlands, patients with SMI-PD tend to be treated in Flexible Assertive Community Treatment (FACT) teams, which are designed to provide community-based care for individuals with SMI. FACT teams employ a flexible switching system that combines care for two patient groups requiring different intensities of support. For clients needing lower-intensity care, individual case management is provided and integrated with multidisciplinary treatment and support. In crisis situations, intensity of care can be escalated, and a shared caseload approach is adopted to ensure outreach, persistent support, and continuity of treatment. The flexibility in switching between the two types of care aims to enhance continuity of care and reduce treatment drop-out (32). Although FACT has been proven effective for patients with SMI, the focus on psychosis as primary patient group limits its applicability for patients with SMI-PD. FACT teams may have less experience addressing the complex needs of patients with PDs, including difficulties related to (counter)transference in the therapeutic relationship, pre-mentalizing modes, and trauma-related symptoms. Consequently, treatment dropout of patients with SMI-PD is rarely attributable solely to intrapsychic, motivational problems; rather, dropout often reflects the patients’ perspective of epistemic mistrust stemming from attachment problems or the inability of conventional care models, such as FACT, to adequately address the full range of potential limitations and challenges associated with SMI-PD (7).

Adaptive Mentalisation-based Integrative Therapy (AMBIT) is a mentalization-based systematic framework that was originally designed to support teams involved in care for young people with multiple needs who are reluctant to trust help (17, 33–35). AMBIT is based on the concept that epistemic mistrust is an undermining factor in the effectiveness of care and treatment, and aims to restore epistemic trust between the individual and their helping network to enable new social learning (18, 33, 34). AMBIT is characterized by adaptability in response to an individual’s needs and that of the systems involved in care around the individual, and to what is perceived as helpful, with a special emphasis on promoting mentalizing skills. Mentalizing is the mental process that allows individuals to interpret and understand the mental states of themselves and others, which underlie overt behaviour (17, 35, 36). AMBIT uses mentalizing as the unifying framework across four domains: in 1) face-to-face interactions with individuals, 2) through structured, team-based mentalizing-enhancing sessions that focus on establishing effective relationships within teams and 3) on collaborating within multi-agency networks and family to care for them, 4) eventually fostering a learning culture. The focus on integrated care and collaboration in a coordinated network around the patient, overlaps with FACT. However, AMBIT differs from FACT, in that AMBIT uses mentalization as its framework and addresses distrust, relational repair, engagement, and adaptability, which eventually restores epistemic trust enabling new social learning. These factors seem to be primary mechanisms of change in AMBIT informed teams and are thought to contribute to the positive and the long-term effectiveness of this approach (33, 37), distinguishing AMBIT from the typical way care for patients with SMI-PD is currently organized such as FACT (6–8). It is suggested that AMBIT facilitates ‘fortuitous events’ by focusing on enabling the helping system surrounding a patient to act in a more understanding (more mentalized) and less conflicted manner. This approach reduces incongruency in the simultaneous support provided to a patient and contributes to the ‘extra-therapeutic change’, ultimately enhancing effectiveness of AMBIT. Findings suggest a generally positive impact of AMBIT-informed teams on symptom reduction and improvements in functioning among individuals with multiple problems (33, 34).

When possible, AMBIT includes evidence-based therapies but also emphasizes practice-based evidence, focusing on what works for the teams involved in supporting the individual. It includes multiple interventions tailored to the specific needs of each individual, which may involve proactive facilitation of access to (and use of) help provided by other agencies (e.g. housing, financial support, courts, education or training, employment support, etc.). These agencies may struggle to engage SMI-PD patients without a “team around” approach, in which a dedicated worker actively mentalizes both their professional predicaments and the patients’ perspective. Such interventions may focus on trauma (e.g., Narrative Exposure Therapy, Eye-Movement Desensitization Reprocessing), family dynamics (systemic therapy or relational therapy), or educational/vocational difficulties (individual placement and support). Mentalization Based Therapy (MBT), an evidence-based psychotherapy for borderline personality disorder (BPD) targeting mentalizing deficits, is typically included for personality difficulties in an AMBIT approach, at least on an individual basis. MBT has demonstrated significant reductions in hospitalization, self-harm, and in improvements in social and interpersonal functioning, with long-lasting benefits (36, 38). Its focus on enhancing mentalizing capacity makes MBT highly likely to be an important component of care for individuals with SMI-PD, making AMBIT particularly suitable for this patient group.

Considering the potential positive impact of AMBIT informed teams on reducing symptoms and improving overall functioning in individuals with multiple problems (33, 39), it is anticipated that this approach reduces epistemic mistrust resulting in more positive experiences in daily life, ultimately breaking the cycle of the persistence of problems. These improvements may also contribute to a reduction in healthcare costs and non-healthcare costs, such as loss of earnings and financial burden because of, amongst others, fewer hospital admissions, fewer crisis contacts, fewer general health costs, reduced time demands on mental health workers, and diminished productivity loss for relatives. A comparative health-economic evaluation is needed to assess whether the AMBIT approach is more cost-effective than FACT.

This study aims to investigate the effectiveness of an AMBIT approach in treatment and care for patients with SMI-PD.

The primary objective of this study is to investigate the effectiveness of AMBIT for patients with SMI-PD in comparison to treatment with FACT. It is hypothesized that an AMBIT approach will be more effective in improving outcomes on a broad spectrum of life domains (e.g. leisure activities, education, housing, family, symptoms, and drug use) in patients with SMI-PD than FACT.

Furthermore, this study aims to investigate differences in treatment effects of the following secondary variables regarding personality functioning, and social- and societal functioning, mentalizing capacity, epistemic trust, and suicidality. It is hypothesized that patients in the AMBIT condition show greater improvement on a broad spectrum of outcomes, considering psychiatric symptoms, social ecology, relationship networks/qualities, and resiliencies as measured by the instruments included in the present study as discussed in the method section.

Cost-effectiveness will also be evaluated for AMBIT. It is hypothesized that AMBIT will be a more cost-effective treatment than FACT.

Lastly this study aims to investigate variables that may moderate and mediate treatment outcome. It is expected that severity of childhood trauma, and duration of illness have a moderating effect on treatment outcome, while epistemic trust and mentalizing capacity are expected to have a mediating effect on treatment outcome.

## 2 Methods

### 2.1 Design

This controlled, naturalistic, longitudinal cohort study will follow participants over 18 months of treatment to assess recovery outcomes in patients with SMI-PD across three settings: FACT, AMBIT-PD, and AMBIT informed-MBT. A naturalistic design was chosen to enhance ecological validity and generalizability, allowing treatment to reflect real-world practice rather than rigid protocols. Unlike a randomized controlled trial (RCT), which prioritizes internal validity, this approach better captures treatment effectiveness in complex, real-life settings where patient motivation—key to therapeutic success— will not be affected by randomization (40–43). Since participants must have already been in mental health care for at least two years (6–8), altering their current care for the sake of an RCT may be unethical and disruptive. The flexibility of the AMBIT approach, which focuses on restoring trust and adapting to individual needs, would also be compromised by a rigid study design, potentially increasing epistemic mistrust and disengagement (42). A naturalistic design minimizes exclusion due to strict criteria and reduces drop-out, leading to more complete data. However, the absence of randomization does increase the risk of bias from unknown confounders, which will be considered in the analysis (40, 42–44).

### 2.2 Participants

The study sample will consist of 160 patients with SMI-PD receiving care at GGZ Rivierduinen and GGZ Altrecht, mental health facilities in the Netherlands. 40 patients will be recruited at the FACT teams of Altrecht (FACT-ALT), 40 patients at the FACT teams of GGZ Rivierduinen (FACT-RIV), 40 patients at the AMBIT-PD teams of Altrecht (AMBIT-PD-ALT) and 40 patients at the AMBIT informed-MBT team of Rivierduinen (AMBIT-MBT-RIV). Participants will be recruited via their principal therapist in each of the participating teams when they meet inclusion criteria. Eligibility of each participant will be assessed before recruitment of a participant.

Participants are eligible if they 1) are 18 years of age or older, 2) are willing and able to give informed consent, 3) have a present diagnosis of a PD meeting DSM 5 criteria, 4) meet the criteria of SMI (5, 7, 8). This includes having a persistent psychiatric disorder that requires care and treatment, having undergone at least two years of treatment without achieving functional recovery (GAF ≤ 50), and experiencing impairment in social and/or societal functioning, such as difficulties in at least two areas of life, including financial issues, educational or occupational challenges, housing problems, relationship issues, substance abuse, or school/work difficulties. The dysfunction must be caused by or result in a psychiatric disorder, and coordinated care from professional caregivers in care networks is necessary to implement a treatment plan.

Participants are excluded from participation in this study if they 1) do not master the Dutch language, 2) are significantly under the influence of substances and are unwilling to detoxify elsewhere, or 3) if they have an intellectual impairment (IQ < 80, based on the Wechsler Adult Intelligence Scale).

#### 2.2.1 Sample size calculation

Based on previous studies reporting reductions in overall psychiatric symptoms following MBT, with effect sizes ranging from 0.59 to 1.79 (38), an *a priori* power analysis was conducted in G*Power 3.1.9.6 to determine the optimal sample size. Assuming a medium effect size (f = 0.25), a significance level of α = 0.05, and 90% power in a repeated-measures ANOVA with three measurements across three conditions, a total of 135 participants would be required to detect an existing effect. Accounting for an anticipated dropout rate of approximately 20%, a final sample size of 162 participants will be recruited to maintain sufficient power.

#### 2.2.2 Feasibility

To map the feasibility of participant recruitment, we rely on the standard guideline of care for personality disorders (45). It is estimated that approximately 30% of the FACT teams meet the criteria for SMI-PD. GGZ Rivierduinen location Leiden has 4 FACT teams. Altrecht location Utrecht has 9 FACT teams. Each team has at least 200 clients in care on a yearly basis. Therefore, approximately 60 clients per FACT team are likely to meet the inclusion criteria of the study, which amounts to around 780 clients (45). As the study is observational in nature and only minimally burdensome for participants (questionnaires), and since we plan to provide a generous participation fee of 30 euros per measurement, we think it is reasonable to expect that around 10% of the clients will consent to participate. From previous naturalistic research (46), it was found that a vast majority (87%) of this target group was willing to participate in research. Rivierduinen has one AMBIT informed-MBT team, whereas Altrecht has five, who each treat 200 to 250 clients, nearly all of whom meet the inclusion criteria of the study, amounting to around 1000 likely eligible participants. Since this also concerns naturalistic research, we are confident that recruiting 80 participants at Rivierduinen and 80 at Altrecht is feasible.

### 2.3 Measures

#### 2.3.1 Main Study parameter/endpoint

The Outcome Questionnaire (OQ-45.2) (47, 48) is a self-report measure designed to monitor overall psychological distress and psychological functioning. The OQ-45.2 consists of 45 items divided into three subscales: subjective distress (intra-psychological functioning), interpersonal functioning, and social role functioning. Items are rated on a 5-point Likert scale ranging from 0 (*never*) to 4 (*almost always*), yielding a total score between 0 and 180. Higher scores indicate greater psychological distress and functional impairment. Total and subscale scores demonstrate adequate reliability and validity (48).

#### 2.3.2 Secondary study parameters/endpoints

##### 2.3.2.1 Demographic variables and clinical data

Several clinical and demographic data will be collected, including sex, age, age of onset of self-harm, education, vocation, days spent in hospitalization due to psychiatric complications, receiving involuntary care under the Compulsory Mental Health Care Act (Wet verplichte geestelijke gezondheidszorg, Wvggz) (49) and number of suicide attempts or self-harm incidents.

##### 2.3.2.2 Personality functioning

The Severity Indices for Personality Problems-Short Form (SIPP-SF) (50) is a self-report questionnaire designed to assess maladaptive personality functioning over the past 3 months. The SIPP-SF consists of 60 items rated on a 4-point Likert scale ranging from 1 (strongly disagree) to 4 (strongly agree). The items measure maladaptive personality functioning across five domains: 1) Self-control, 2) Identity integration, 3) Relational capacities, 4) Responsibility, 5) Social concordance. Higher scores in each domain indicate more adaptive functioning, whereas lower scores reflect greater maladaptive traits. The SIPP-SF is a valid and reliable measure for maladaptive personality characteristics (50).

##### 2.3.2.3 Social and societal functioning

The Global Assessment of functioning (GAF) is a clinician-rated scale ranging from 0 to 100 that evaluates psychosocial, occupational, and social functioning. Higher scores indicate better overall functioning, whereas lower scores reflect more severe impairment. The GAF provides a standardized assessment of how symptoms affect an individual’s daily life. The scale has demonstrated adequate reliability and validity (51).

The AMBIT Integrative Measure (AIM, paper version or interactive version) (52) is based on the Hampstead Child Adaptation Measure (HCAM) and the Functional Assessment of Care Environments (FACE) (52), and evaluates outcomes based on changes in areas most relevant to the individual, rather than solely relying on diagnostic outcomes. Its aim is to capture meaningful improvements in daily life, social relationships, and overall psychosocial functioning as experienced by the individual. The measure consists of 43 items across 8 domains of functioning: Daily life, Socio-economic factors, Family relationships, Social relationships, Mental states, Response to situation, Complexity, Power and Control. Items are rated on a 5-point scale ranging from 0 (*no problem*) to 4 (*severe problem)*, with reductions in scores indicating improvement. The AIM is designed to be low-burden and practical for routine use, completed both by the practitioner (case manager in the FACT team or key worker in the AMBIT team) and by a blinded rater. The measure demonstrates good reliability and validity (52).

##### 2.3.2.4 Mentalizing capacity

The Reflective Functioning Questionnaire (RFQ) (53) is an 8-item self-report measure of mentalizing that captures an individual’s perceived capacity to understand their own and others’ mental states. It includes two subscales: Certainty (hypermentalizing) and Uncertainty (hypomentalizing). Items are rated on a 7-point Likert scale ranging from 1 (*strongly disagree)* to 7 (*strongly agree*). Higher scores represent hypo-mentalizing whereas lower scores represent more optimal mentalizing. The measure shows good reliability and validity (53).

##### 2.3.2.5 Epistemic mistrust

The Questionnaire Epistemic Trust (QET) (54) is a 24-item self-report questionnaire designed to assess epistemic (mis)trust in interpersonal contexts. It consists of 4 subscales: 1) Hypervigilance, 2) Curiosity/Openness, 3) Expectations of Help, 4) Openness to help. Items are rated on a 5-point Likert scale varying from 1 (*totally agree*) to 5 (*totally disagree*). Total scores range from 24 to 120, with higher scores indicating greater epistemic mistrust. The subscales have good to excellent internal consistency and an excellent internal consistency for the total 24-item scale (Cronbach’s alpha α = 0.91). The QET has shown relevant associations with related constructs like personality functioning, symptom distress and quality of life (54).

##### 2.3.2.6 Childhood trauma and attachment security

Childhood trauma and adversity will be assessed using the Childhood Trauma Questionnaire (CTQ) (51, 55–57), and the Youth and Childhood Adversity Scale (YCAS) (58). The CTQ is a 28-item self-report measure assessing severity of childhood abuse and neglect, and includes five subscales: 1) Emotional Abuse, 2) Physical Abuse, 3) Sexual Abuse, 4) Emotional Neglect, and 5) Physical Neglect. Items are rated on a 5-point Likert scale, ranging from 1 (*never true*) to 5 (*very often true*), with higher scores indicating greater severity. The questionnaire includes a minimization/denial scale to identify potential under-reporting of trauma, enhancing measurement accuracy and reliability. The CTQ demonstrates good reliability and validity across (clinical) populations (57).

The YCAS is a 13-item retrospective self-report questionnaire assessing exposure to adverse life events (e.g., serious illness, serious parental illness, or “other” adversity). Participants need to indicate whether they have experienced each type of adversity (yes/no). For any endorsed event, participants need to rate the perceived severity on a 7-point scale from 1 (*not at all traumatic*) to 7 (*extremely traumatic*). The YCAS has good reliability and validity (58).

Autonomy and attachment will be assessed using the Autonomy-Connectedness Scale (ACS-30) in Dutch (59, 60). The ACS-30 consists of 30 items across 3 subscales: 1) Self-awareness (awareness of one’s own opinions, desires, and needs during social interactions), 2) Sensitivity to others (empathy and consideration of others’ opinions), 3) Ability to handle new situations (comfort with unfamiliar situations, flexibility, and exploration). Items are rated on a 5-point Likert scale from 1 (*strongly disagree*) to 5 (*strongly agree*), with higher scores indicating greater autonomy and secure attachment. The measure demonstrates good reliability and validity (59).

##### 2.3.2.7 Suicidality

The Columbia-Suicide Severity Rating Scale (C-SSRS) (61) is an instrument that screens for suicidal ideation and behavior across four domains: 1) severity of ideation (5 items), 2) intensity of ideation (5 items), 3) suicidal behavior (5 items), and 4) lethality subscale (1 or 3 item(s)). Severity of suicidal ideation is rated on a 5-point ordinal scale: 1= wish to be dead, 2 = nonspecific active suicidal thoughts, 3 = suicidal thoughts with methods, 4 = suicidal intent, and 5 = suicidal intent with plan, with higher scores indicating more severe ideation. Intensity of ideation is rated on a 5-point ordinal scale assessing frequency, duration, controllability, deterrents, and associated distress, with higher scores indicating greater intensity. Suicidal behavior is rated on a nominal scale including actual, aborted, and interrupted attempts; preparatory behavior; and nonsuicidal self-injurious behavior. Lethality is rated on a 6-point ordinal scale for actual lethality, or a 3-point scale for potential lethality if actual lethality is zero. The C-SSRS demonstrates good reliability and validity (61, 62).

The SUICIdal DIfferentiation (SUICIDI) version 3.2 (63) is a clinician-rated instrument designed to differentiate types of suicidality based on the four-type model of entrapment (4ME) (63–65). The SUICIDI assesses four subtypes: 1) Perceptual Disintegration (PD), 2) Primary Depressive Cognition (PDC), 3) Psychosocial Turmoil (PT), and 4) Inadequate Coping or Communication (IC). A total of four points is distributed across these subtypes, reflecting the relative prominence of each in the patients’ presentation. Higher scores within a subtype indicate stronger alignment with that specific pattern of suicidality. Scores can be divided among subtypes when uncertainty exists, but the rater must determine one dominant subtype based on overall clinical impression. The instrument is completed by a trained clinician or caseworker following a structured interview or case review. The SUICIDI demonstrates good to excellent interrater agreement (63).

##### 2.3.2.8 Cost-effectiveness

Health-related quality of life will be assessed using the Dutch version of the EQ-5D-5L (66). The EQ-5D-5L is a self-report measure that evaluates health across five dimensions: mobility, self-care, usual activities, pain/discomfort, and anxiety/depression. Each dimension is rated on a 5-point scale ranging from 1 (*no problems*) to 5 (*extreme problems/unable to*), generating a health state that can be converted into a utility score (67). The Dutch tariff (68), will be used to derive utility values from EQ-5D-5L responses, from which quality-of-life adjusted life years (QALYs) will be calculated over the 3 years follow-up period. One QALY represents a year lived in a state of perfect health, combining both quality and length of life into a single metric. Symptom reduction and improvements in daily functioning are expected to increase QALYs, resulting in a dominant incremental cost-utility ratio (greater QALY health gains for lower healthcare costs and non-healthcare costs). These findings would support AMBIT as a more cost-effective treatment compared with treatment as usual (i.e., FACT) (69).

Individual capability will be assessed using the Dutch version of the ICECAP-A (70–72). The ICECAP-A measures five capabilities relevant to quality of life: stability, attachment, autonomy, achievement, and enjoyment. Each of five items is rated on a 4-point Likert scale ranging from 1 (not able to experience capability at all) to 4 (fully able to experience capability). Responses will be transformed into a capability index score using a tariff based on the Dutch general population. Index scores range from 0 (no capability) to 1 (full capability). The ICECAP-A demonstrates good test-retest reliability (ICC = 0.79) and construct validity in a large Dutch population (72), and has acceptable levels of content validity (70, 73).

Healthcare costs will be measured using the Treatment Inventory of Costs in Patients with psychiatric disorders (TiC-P) Midi (74), a self-report instrument consisting of 8 items. The TiC-P will be administered at each assessment and captures costs related to both intervention costs and use of health care services. These costs cover outpatient services, including therapy costs for both treatment as usual (TAU, i.e., FACT) and AMBIT programs, medical specialist and paramedic visits, home care assistance, and medication.

##### 2.3.2.9 State resilience and trait resilience following childhood trauma

Psychosocial resilience refers to an individual’s ability to adapt, recover, and maintain well-being in the face of stress, adversity, or challenges in their social and psychological environment (75, 76). Levels of resilience vary across individuals depending on the presence and interaction of protective psychological, emotional, and social factors. These protective factors are considered to facilitate coping with stressors, thereby reducing the risk of developing mental health problems (76). Resilience can best be understood and measured as a dynamic process of adaptation and recovery during and following stressors (75, 77).

Resilience during a stressor, i.e. state resilience, can be measured as daily life psychosocial resilience in response to momentary stressors. The use of momentary assessments offers a more dynamic, real-time perspective on how people cope with stress in their daily lives. Daily life state psychosocial resilience is assessed using an electronic diary through the Experience Sampling Method (ESM) via the PsyMate smartphone app (78, 79). Participants will complete brief questionnaires 10 times per day over 5 consecutive days. Items assess activity-related stress (“*I find the activity I am currently doing difficult*”) and event-related stress (“*I just experienced a difficult event*”), as well as social context (alone or with others), social stress (e.g., “*I would rather be alone*”), social support (e.g., “*I like the present company”)*, and emotional state (positive and negative). Negative emotions are calculated as the average of responses to feelings such as “*anxious*,” “*lonely*,” “*insecure*,” “*irritated*,” “*down*,” “*guilty*,” and “*gloomy*”, whereas positive emotions are calculated from “*happy*,” “*satisfied*,” “*cheerful*,” “*relaxed*,” and “*enthusiastic*”. All items are rated on a 7-point Likert scale ranging from 1 (*not at all*) to 7 (*very much*). Daily life psychosocial resilience is conceptualized as a decrease in negative emotions and an increase in positive emotions in response to activity-, event- or social-related stress.

Resilience following stressors, such as childhood trauma, provides a broader assessment of an individual’s current mental health and well-being in the aftermath of early adverse experiences. Trait psychosocial resilience is quantified using the residual variance method (80–82), a statistical approach that operationalizes resilience as the extent to which an individual functions better (or worse) than expected given their level of experienced stress or adversity. Specifically, resilience is calculated as the individual residual variance from the population average, based on a regression of current mental health functioning on childhood experiences. This method provides an objective, quantitative measure of resilience and allows examination of how both intrinsic and external factors contribute to an individual’s capacity to cope with stressors. Trait resilience is assessed using a combination of mental wellbeing instruments (e.g., GAF, AIM, C-SSRS) and self-reported childhood adverse events (e.g., CTQ, YCAS) (80–82).

##### 2.3.2.10 Keyworker/Case manager work satisfaction

The Burnout Assessment Tool (BAT) (83) is a 23-item self-report questionnaire to measure burn-out. It includes four dimensions: 1) exhaustion (EX; 8 items), 2) mental distance (MD; 5 items), 3) cognitive impairment (CI; 5 items) and 4) emotional impairment (EI; 5 items). The items are rated on a 5-point Likert scale, ranging from 1 (*never*) to 5 (*always*), with higher scores indicating higher levels of burnout. The Dutch version of the BAT has sound psychometric properties (83, 84).

##### 2.3.2.11 Qualitative interview: the significance of treatment

At the end of the study (T2), a qualitative interview will be conducted with 20 participants to determine what patients themselves found to be most conducive or detrimental to their personal recovery in their respective treatment approaches. A qualitative approach was chosen for this study to “enter into the world of participants, to see the world from their perspective, and in doing so make discoveries” (78). To explore the significance of either FACT or AMBIT treatments in the recovery of patients, in-depth semi-structured interviews will be conducted based on grounded theory. All interviews will be transcribed verbatim. The transcripts will be analysed using the qualitative data analysis program ATLAS.TI (Version 24, VERBI Software, 2022), following a grounded theory approach. In the “open coding” phase, interview data will be broken down into smaller components, “codes,” which are specific concepts or themes identified in the data. As data is coded, the researchers continuously compare new data with previous codes and categories. After open coding, related codes are grouped into broader categories. Axial coding is the process of identifying relationships between these categories, which helps in understanding the structure of the emerging theory. As the analysis progresses, the researcher focuses on a core category that seems central to the data. This core category will become the foundation of the emerging theory. Selective coding involves refining and integrating categories around this core category to form a cohesive theory. Researchers will continue to collect data until “theoretical saturation” is reached—when no new information or insights are being discovered, and the categories are well-developed. Sampling decisions are guided by the emerging theory rather than by a predetermined plan. Throughout the process, researchers will write memos to capture their thoughts, hypotheses, and reflections on the data and emerging theory. Memos are a crucial tool for developing the theoretical framework. Theory Development: The final theory is grounded in the data, meaning it is directly derived from the participants’ experiences and the researcher’s analysis.

### 2.4 Study procedures

After recruitment, names of interested individuals will be communicated to the researchers of the study, who will then invite each potential participant for a meeting during which screening will take place and information on the study will be provided. When a potential participant is eligible and agrees to participate in the study, an informed consent must be signed. Eligible participants who have given informed consent will receive an email containing a notification of eligibility, explanation of the study, and an invitation for the baseline assessment (T0).

The baseline assessment will be held at the department where treatment takes place, which will also minimize the risk of dropout due to missed assessment appointments.

Follow-up assessment will take place during treatment, at 9 months (T1) and 18 months (T2). After 18 months, participants will continue their treatment if this is still needed, but study participation will end. Participants will be asked if they are willing to participate in assessment 5 years after the study is completed. **Table 1** presents the measurements used in each stage of the study.

**Table 1:** Overview of assessments and their content.

Instrument	Time to complete	T0 Baseline	T1 After 9 months of treatment	T2 After 18 months of treatment, end of study participation
Instruments administered to the participant				
OQ-45.2	5 minutes	X	X	X
SIPP-SF	15–20 minutes	X	X	X
RFQ	3 minutes	X	X	X
QET	5 minutes	X	X	x
CTQ	5 minutes	X	–	–
YCAS	2 minutes	X	–	–
C-SSRS	2 minutes	X	X	X
ACS-30	15 minutes	X	X	X
ESM	2 minutes 10 times a day, for 5 consecutive days	X	X	X
EQ-5D-5L	5 minutes	X	X	X
ICECAP-A	5 minutes	X	X	X
TiC-P	30 minutes	X	X	X
Qualitative interview	60 minutes	–	–	X
Instruments administered to the therapist/case worker				
Demographics and clinical data (GAF)	Not relevant	X	–	–
AIM	Not relevant	X	X	X
SUICIDI-3.2	Not relevant	X	X	X
BAT	Not relevant	X	X	X
Receiving involuntary care	Not relevant	X	X	X

### 2.5 Treatment conditions

**Table 2** present an overview of the overlapping and distinct components of the different treatment conditions.

**Table 2:** Overview of the overlapping and distinct components of the different treatment conditions.

Component	FACT	AMBIT	MBT
Integrated care (e.g. shared caseload)	+	+	–
Collaboration in coordinated network around patient	+	+	–
Addressing distrust, relational repair, engagement, adaptability	–	+	+
team-based mentalizing-enhancing sessions	–	+	+
Main patient group	Patients with psychosis	Patients with PD	Patients with PD

#### 2.5.1 Adaptive mentalization-based integrative therapy

AMBIT informed teams share a mentalized approach to integrate care provided to their patients but may differ in the kind of interventions they offer in meeting the needs of their patients. In this study participants can either be assigned to an AMBIT informed-MBT team (Rivierduinen) or to an AMBIT-PD team (Altrecht). Mentalization based treatment is an important component in both AMBIT conditions (whether AMBIT informed-MBT or AMBIT-PD) by focusing on strengthening an individual’s ability to mentalize—reflecting on mental states in oneself and others—especially within emotional relationships.

##### 2.5.1.1 AMBIT informed-MBT

The AMBIT informed-MBT condition is provided at Rivierduinen. The standard interventions offered to patients in this condition consist of MBT treatment within an AMBIT framework, including group therapy one to three times per week and one individual session weekly. If required to meet the needs of a patient, family therapy, trauma therapy (either EMDR or Narrative Exposure Therapy), mindfulness, psychodrama, pharmacotherapy, or support in areas of education, career, and housing may be included. When experience is not available within the team, emphasis is placed on seeking experience outside the team. The MBT methodology is applied/executed by trained therapists over 18 months, focusing on developing mentalizing capacity, regulating emotions, and restoring relationships. The team also received training and supervision from the Anna Freud Centre London to work according to the AMBIT methodology with the aim of building a more mentalizing network around the patient. Intervision regarding MBT and AMBIT is provided on a weekly basis.

##### 2.5.1.2 AMBIT-PD

The AMBIT- PD condition is provided in five teams at Altrecht. These teams are trained in the AMBIT methodology and focus on working in a mentalized manner with patients and aim at building a mentalizing network around the patients. These teams have daily multidisciplinary meetings and every two weeks either InterVision or supervision with the team. A major difference with the AMBIT informed MBT condition is that MBT group-therapy is not mandatory in the AMBIT-PD condition. In the AMBIT informed MBT condition, therapy is individualized, meaning that whether patients receive MBT group-therapy, individual MBT and other interventions, such as, family therapy, pharmacotherapy or trauma-therapy is adjusted to the patient’s needs and wishes. Patients in these teams are treated for, on average, three to five years, but sometimes longer.

#### 2.5.2 Functional assertive community treatment

Participants in the Functional Assertive Community Treatment (FACT) teams receive a versatile treatment based on the ‘Functional Assertive Community Treatment’ model. Treatment in FACT teams includes outreaching care, pharmacotherapy, case management and psychoeducation, but can also provide psychological interventions (Cognitive Behavioural Therapy or EMDR) when indicated (32). FACT teams are present both at Rivierduinen and Altrecht.

### 2.6 Therapists and adherence

AMBIT informed treatment in the different AMBIT teams will be conducted by a multidisciplinary team consisting of psychologists with varying degrees of clinical education and experience, psychotherapists, psychiatrists, social nurses and an expert patient. Most of the team members are AMBIT informed by trainers of the Anna Freud Centre in London. Most of the team members are also trained in MBT and other psychotherapies such as EMDR. Adherence to the AMBIT approach will be monitored through supervision by the Anna Freud Centre and through InterVision within the team.

FACT treatment is provided in a multidisciplinary team consisting of psychologists with varying degrees of clinical education and experience, at least one psychiatrist, psychiatric nurses, social workers, psychologists, peer support workers, IPS workers. Education and supervision are provided on a regular basis to ensure expertise and quality in working according to the FACT model, which will also ensure treatment as usual for participants assigned to this condition (control group). Patients in this group represent the control group and receive treatment as usual.

### 2.7 Monitoring participants’ response and follow-up on assessment

The present study involves severely psychiatrically ill patients with a reduced life expectancy, among whom attempts and even suicides may also occur. Due to the generally higher suicide risk of our patients, an attempt or even suicide may take place independently of the intervention. Any occurrences of severe suicidal behaviour will be thoroughly analysed to assess whether there were any shortcomings in the provided care. The assessment in this study therefore includes questionnaires and questionnaire items that focus on suicidality to identify the type of suicidality to better prevent suicidal behaviour among this group of patients. Even though asking about suicidality does not increase suicidal risk (85), the participants’ response on the assessment must be monitored and followed up. The procedure below will be followed while and after assessment:

- Scores and responses on questionnaires and questionnaire items that focus on suicidality must be examined before the participant leaves the research setting.
- In case suicidality is endorsed through responses to suicide-relevant questionnaires or items, and it was not previously known or there is a clinical impression that it has increased in severity, the researcher must immediately respond to the participant by acknowledging the presence of his or her suicidal ideations or behaviour and informing the participant that he or she will be contacted by the principal therapist involved in treatment of the participant.
- The researcher must inform the principal therapist involved in treatment of the participant, who will reach out to the participant on the same day (< 24 hours) the assessment has taken place.
- If an imminent risk is present, the researcher must remain with the participant until support is received.
- Guidelines will be followed in how the involved caregivers anticipate the participants’ suicidality.
- Additionally, an independent psychiatrist specialized in suicidality and the legal circumstances surrounding this concept can be consulted. There is also a close relationship with a health law expert with whom risks can be discussed.

The monitoring and follow-up procedures after assessment are explained during the first meeting in which study information is provided and informed consent is obtained. These procedures are also included in the informed consent given to the participant.

Researchers involved in this study are trained in how to deal with suicidality by the department (Suicide prevention basic-/refresher course). This training is mandatory for everyone involved in patient treatment at Rivierduinen and Altrecht.

### 2.8 Data monitoring and management

Data of participants will be handled and saved strictly confidential according to the Declaration of Helsinki and national regulations of the Dutch Ethics Committees on research involving humans. Study data will be entered in an SPSS data file and saved on a secured server of GGZ Rivierduinen. It will be accessible (under restricted access) after the articles have been published and can be requested via the principal researcher. Consequently, a decision will be made about the request, and the following points will be considered: data security, the period of approval for use of the data file, the way in which the data file will be accessible, collaboration when the data file is used including arrangements about publication and authorships.

Adverse events will be documented by the research team and reported immediately to the Commissie Wetenschappelijk Onderzoek (CWO). All adverse events will be followed until they have abated, or until a stable situation has been reached.

### 2.9 Ethical considerations

This study will be conducted in accordance with the Declaration of Helsinki and national regulations of the Dutch Ethics Committees on research involving humans. Ethical approval for this study was obtained METC Leiden Den Haag Delft, reference number N24.090, and the study protocol was peer reviewed. The study was preregistered on May 7, 2025, at OSF Registries, https://doi.org/10.17605/OSF.IO/QHYCW. The study was registered at Research Portal of CCMO Onderzoek met mensen, Trial Registration Number NL-010303, https://www.onderzoekmetmensen.nl/en/trial/57877, June 27, 2025, which is approved data provider of the International Clinical Trials Registry Platform of the World Health Organisation. All participants have to give informed consent for participating in the study.

Participants can leave the study at any time for any reason if they wish to do so without any consequences for their care or treatment at GGZ Rivierduinen or Altrecht. Recruitment of subjects will continue until the needed number of participants to complete the study is attained. There are no anticipated risks for taking part in this study. The study consists of the completion of self-report questionnaires and of keeping a diary. Every participant will get treatment as was indicated based on his or her individual needs.

## 3 Statistical analyses

### 3.1 Primary analyses

Data will be analysed using intention-to-treat analysis repeated measures analysis. Missing data due to drop-out will be imputed using a Monte Carlo Markov chain with 5000 iterations.

#### 3.1.1 Baseline differences and matching

Differences in baseline demographic and clinical characteristics between participants in the AMBIT informed and FACT conditions will be assessed using appropriate statistical models. For dichotomous variables, chi-square tests or Fisher’s exact tests (where applicable) will be conducted, while continuous variables will be analysed using independent samples t-tests or Mann-Whitney U tests in the case of non-normal distributions. Additionally, multivariate logistic regression (for categorical outcomes) and linear regression (for continuous outcomes) will be employed to adjust for potential confounders and assess the robustness of group differences.

The total sample of participants will be divided in different groups, based on identified clinically relevant differences in baseline demographic or clinical characteristics. Patient pairs will be matched on the smallest difference in propensity score (see below) within these groups (44).

#### 3.1.2 Propensity score matching

A propensity score is calculated, which is a statistical approach that attempts to reduce selection bias and known confounding that could be found in an estimate of treatment outcome in an observational study. A propensity score is the conditional probability that a participant in a study will be assigned to one of two treatment groups in this study, given a set of observed pre-treatment characteristics. Pre-treatment characteristics are demographic and clinical characteristics of participants (partly) determining the indication for a specific treatment, such as diagnoses or social situation. They will be included in the propensity score calculation as they may be related to treatment outcome and are therefore considered potential confounders. A linear regression analysis will be conducted to identify how pre-treatment variables relate to outcome, with pre-treatment variables as the dependent variable and each potential confounder as an independent variable. The propensity score for each participant will then be estimated using logistic regression analysis with all potential confounders as independent variables and ‘group membership’ (the condition to which the participant was assigned to) as the dependent variable. Each participant from one condition will then be matched with a participant from the other condition, based on the nearest available propensity score, pairs with a propensity score difference of 0.10 will be removed from the analysis, resulting in matched pairs that show no difference on any of the observed pre-treatment characteristics. A regression analysis will then be conducted in the matched sample with the identified confounding pre-treatment characteristics as dependent variable and the ‘group membership’ as the independent variable to reduce the effect of treatment group membership (44, 86, 87).

#### 3.1.3 Multilevel (mixed) model analysis

Multilevel (mixed) model analysis will be conducted to compare outcomes for both conditions on the OQ-45.2, with baseline scores (T0), scores after 9 months (T1), and at the end of treatment after 18 months (T2) as dependent variables and treatment (FACT or AMBIT) as independent variable. The different levels for the multilevel (mixed) model analysis will be: 1) participant, 2) measurement wave, 3) outcome measure, and 4) location of sampling. A multilevel (mixed) model analysis uses all data and contributes to power in the analysis in contrast to case wise deletion for missing data.

#### 3.1.4 Moderating and mediating effects

To analyse the moderating effect of the factor variable (i.e. severity of childhood trauma and duration of illness) on treatment outcome in both treatment conditions an ANOVA will be performed, with the moderating factors, treatment condition and their interaction term as independent variables and the OQ-45.2 scores as dependent outcomes.

A linear regression analysis will be performed to investigate a potential mediating relationship between the treatment conditions and epistemic trust and reflective functioning.

Furthermore, given potential differences across locations, an exploratory moderation analyses with location as a categorical moderator will be conducted.

### 3.2 Secondary analyses

As for the primary analysis multilevel (mixed) models will be used for evaluation of the course of the ESM outcome variables over time. Since ESM data of each questionnaire is nested within days and again within the measurement period (T0, T1 or T2), measurements cannot be deemed independent, which requires the analysis of the data within a multilevel structure. The different levels for the multilevel (mixed) model analysis will be: 1) participant, 2) measurement wave, 3) outcome measure, and 4) location of sampling.

In order to compare within group differences (AMBIT informed-MBT vs AMBIT-PD) in the AMBIT condition on the primary outcome, multilevel (mixed) model analyses will be conducted to, with baseline scores (T0), scores after 9 months (T1), and at the end of treatment after 18 months (T2) as dependent variables and treatment (AMBIT informed-MBT and AMBIT-PD) as independent variable.

### 3.3 Cost-effectiveness analyses

#### 3.3.1 Cost-analysis

The TiC-P Midi will be utilized to quantify costs associated with patient care and productivity losses. The TiC-P will be adapted for this study to accurately reflect healthcare consumption in the target population and the treatment received. The units of healthcare consumption (e.g., contacts, sessions, hospital days) will be multiplied by their full economic cost-price as published in the appendix of the Dutch guideline for costing in healthcare (Zorginstituut Nederland, 2016 or later when an update becomes available). Medication costs will be calculated based on the Daily Defined Dosage (www.medicijnkosten.nl). All costs will be indexed to the reference year 2018. Cumulative costs over 9- and 18-month follow-up periods will be calculated using the area under the curve method. Costs (and effects) will be discounted as appropriate when exceeding the time horizon of 12 months and the discount rates will be subject to sensitivity analysis.

#### 3.3.2 Cost-effectiveness analyses

The health-economic evaluation will be conducted using three approaches: 1) cost-minimization analysis (CMA), 2) cost-effectiveness analysis (CEA), and 3) cost-utility analysis (CUA). Analyses will be performed from both the healthcare system and societal perspectives (cf. Zorginstituut Nederland, 2024). Four types of costs will be considered: 1) treatment costs, 2) costs stemming from additional healthcare uptake, 3) patients’ and family out of pocket costs, 4) costs of productivity losses stemming from absenteeism and reduced efficiency at work (presenteeism). Cost data will be collected using the TiC-P, with standard cost prices obtained from Zorginstituut Nederland (2024 or later) and indexed to the year 2025. The CMA will focus on the cost difference between the conditions, specifically with regard to treatment, healthcare and societal costs. The primary clinical endpoint for the CEA will be incremental costs per reliable change over 18 months. The reliable change for AIM scores between T0 and T3 over 18 months will be calculated using the Jacobson and Truax formula (88). For the CUA, utility values derived from the EQ-5D-5L will be used to compute cumulative QALY gains over the first 9 months and the full 18-month follow-up. The propensity weights (see Analysis) will be incorporated into the cost-effectiveness and cost-utility analyses (CEA and CUA) using inverse propensity score-weighted seemingly unrelated regression equations (SURE) models to simultaneously estimate incremental effects and costs. The SURE models can be bootstrapped (5000 times) to produce graphs such as the scatter of simulated ICERs over the ICER plane and to produce the cost-effectiveness acceptability curve for further probabilistic inference and decision-making purposes. Sensitivity analysis will be directed at the main cost-drivers. The CMA, CEA and CUA will be reported according to CHEERS statement.

Incremental cost and effectiveness of AMBIT compared with treatment as usual (TAU) will be calculated for the CEA. Incremental costs are defined as the mean difference between AMBIT and TAU in total costs over 3 years. Incremental effectiveness is the mean difference in the scores on the measurement instruments over 3 years. For the CUA, incremental cost-utility will be calculated as the difference in total costs divided by the difference in QALYs.

## 4 Discussion

The present study aims to investigate the effectiveness and cost-effectiveness of AMBIT informed-MBT, AMBIT-PD and FACT for patients with SMI-PD, compared to existing treatment in FACT teams. Scientific research is particularly important for this population, as they experience significant distress and frequently do not receive adequate care within conventional treatment pathways. This study aims to explore differences in treatment effects on global functioning, mentalizing capacity, epistemic trust, suicidality and cost-effectiveness. Additionally, it seeks to identify mediators and moderators of treatment outcomes to better understand factors that contribute to therapeutic success.

This study design has several strengths. First, it aims to contribute to the development of AMBIT-informed care tailored to the individual needs of patients with SMI-PD. AMBIT is based on the concept that epistemic mistrust can undermine effectiveness of care and treatment, and focuses on mentalizing, an integrative approach, collaboration and network building, addressing distrust and engagement, and adaptability. Second, the current study has a controlled naturalistic longitudinal cohort design, offering the possibility to adapt treatment and circumstances to the participants included in the study in such a way that it stays close to everyday practice of care for these patients. It can therefore be considered a more ecologically valid and generalizable approach to effectiveness research in psychotherapy. Finally, to our knowledge, (cost-)effectiveness of an AMBIT-informed treatment has not yet been investigated but is important for determining which intervention may be preferred.

Some limitations should also be considered in the current study. First, the overall effectiveness of an AMBIT approach may be limited due to the challenges in the organization and implementation of the model. Second, although a naturalistic design with a control group enables more generalizable comparisons of effectiveness across different treatment settings for patients with SMI-PD and yields more ecologically valid data (40–44), it also introduces a risk of bias due to unmeasured confounders and the absence of randomization. However, this type of design carries the risk of bias due to the presence of unknown confounders resulting from the absence of randomization. The relatively low internal validity and efficacy associated with this type of design represents additional potential sources of bias (40, 42, 43). Lastly, less-time consuming self-report measures were used to minimize participants’ burden while still capturing relevant outcomes. Although the selected questionnaires have been extensively validated and show good psychometric properties, the use of self-report instruments (e.g., RFQ) may be limited by impaired self-reflection, social desirability, or response biases (89–91). These possible biases should be considered in the analysis and interpretation of data. To provide a broader perspective on functioning, clinician-based assessments were included.

Little is known about the treatment needs of patients with SMI-PD and the effectiveness of an AMBIT approach for this group of patients. Furthermore, to our knowledge, the cost-effectiveness of an AMBIT approach in treating patients with SMI-PD has not yet been investigated. The findings of the present study will contribute to the development of tailored and effective treatment for this complex group of patients, who experience high levels of distress and poor quality of life, while often being difficult to engage in healthcare due to their epistemic mistrust.
